# Estimating the Costs of Implementing Comprehensive Primary Care: A Narrative Review

**DOI:** 10.1177/2333392819842484

**Published:** 2019-04-30

**Authors:** Grant R. Martsolf, Ryan Kandrack, Mark W. Friedberg, Brian Briscombe, Peter S. Hussey, Christiane LaBonte

**Affiliations:** 1 RAND Corporation, Pittsburgh, PA, USA; 2 Department of Acute and Tertiary Care, School of Nursing, University of Pittsburgh, Pittsburgh, PA, USA; 3 Department of Health Policy and Management, Gillings School of Global Public Health, University of North Carolina at Chapel Hill, Chapel Hill, NC, USA; 4 RAND Corporation, Boston, MA, USA; 5 Division of General Internal Medicine, Brigham and Women’s Hospital, Boston, MA, USA; 6 Department of Medicine, Harvard Medical School, Boston, MA, USA; 7 RAND Corporation, Washington, DC, USA; 8 Centers for Medicare and Medicaid Innovation, Baltimore, MD, USA

**Keywords:** primary care, medical cost, patient-centeredness, health economics, practice management

## Abstract

The performance of the any health-care system relies on a high-functioning primary care system. Increasing primary care practices’ adoption of “comprehensive primary care” capabilities might yield meaningful improvements in the quality and efficiency of primary care. However, many comprehensive primary care capabilities, such as care management and coordination, are not compensated via traditional fee-for-service payment. To calculate new payments for these capabilities, policymakers would need estimates of the costs that practices incur when adopting, maintaining, and using the capabilities. We performed a narrative review of the existing literature on the costs of adopting and implementing comprehensive primary care capabilities. These studies have found that practices incur significant costs when adopting and implementing comprehensive primary care capabilities. However, the studies had significant limitations that prevent extensive use of their estimates for payment policy. Particularly, the strongest studies focused on a small numbers of practices in specific geographic areas and the concepts and methods used to assess costs varied greatly across the studies. Furthermore, none of the studies in our review attempted to estimate differences in costs across practices with patients at varying levels of complexity and illness burden which is important for risk-adjusting payments to practices. Therefore, due to the heterogeneous designs and limited generalizability of published studies highlight the need for additional research, especially if payers wish to link their financial support for comprehensive primary care capabilities to the costs of these capabilities for primary care practices.

## Inquiry Submission Questions

1. What do we already know about this topic? We know that payers are experimenting with payment models for comprehensive primary care.
2. How does your research contribute to the field? We know little about much payments rates should be set within these models because we do not know how much it costs practices to deliver comprehensive primary care; our paper reviews the literature related to these costs.
3. What are your research’s implications toward theory, practice, or policy? Due to the heterogeneous designs and limited generalizability of published studies highlight the need for additional research, especially if payers wish to link their financial support for comprehensive primary care capabilities to the costs of these capabilities for primary care practices.

## Introduction

The American health-care system is characterized by high costs and uneven quality, due in part to care fragmentation and payment systems that incentivize the utilization of low-value services. Evidence suggests that strengthening the primary care system could yield meaningful improvements in the quality, efficiency, and coordination of care in the United States.^1^ To this end, emerging primary care delivery models focus on helping primary care practices develop and implement capabilities that support “comprehensive primary care,”^2^ which for simplicity we use interchangeably with related concepts, such as advanced primary care and patient-centered medical home models.

Comprehensive primary care models include patient services above and beyond face-to-face visits that can be billed to payers through evaluation and management and preventive care visit codes. For example, comprehensive primary care practices are encouraged to adopt capabilities, such as care management, care coordination, expanded access including after-hours visits and electronic communication with providers, and advanced health information technology.^3–6^ Yet, because fee-for-service payment systems generally only pay practices for direct face-to-face patient care, practices that provide comprehensive primary care capabilities might do so without direct reimbursement from payers.^7^

To promote comprehensive primary care, the Centers for Medicare and Medicaid Services (CMS) and other payers are experimenting with new primary care payment models.^8,9^ These payment models typically incorporate a combination of monthly care management fees, visit-based fee-for-service payments, and performance-based payments tied to quality and efficiency goals.^9^ To avoid over- or underpaying for comprehensive primary care capabilities, payers need accurate estimates of the costs that practices incur when adopting and delivering comprehensive primary care.

We performed a narrative review of empirical research studies that have attempted to estimate the costs that practices incur when adopting and delivering comprehensive primary care. We then discuss important limitations of that literature and discuss how it provides guidance on related primary care payment issues.

## Review Methods

To identify relevant literature, we used a directed reference mining approach. First, we reviewed a recent Agency for Healthcare Research and Quality (AHRQ) report^10^ that outlined 15 AHRQ-funded projects that aimed to estimate the costs of medical home transformation and included 5 published studies resulting from these projects.^11–15^ We then mined the references of those 5 studies and used PubMed and Google Scholar to search for articles that have cited those studies. We excluded studies that estimated costs incurred by a health plan on behalf of a practice, or focused on care settings other than primary care practices. After exclusions, this search yielded 8 studies.

## Costs Incurred by Practices Delivering Comprehensive Primary Care

In this section, we review the findings of the 8 studies estimating the costs that practices have incurred when adopting and delivering comprehensive primary care capabilities. We describe the methods that the researchers used to collect data, the types of costs collected, and the results of the studies. We present a summary of the approaches and findings in Table 1. In the table, we differentiate start-up versus annual ongoing costs. Where possible, we estimate annual costs as total, per clinician, and per patient.

**Table 1.** Summary of Study Methods and Findings.

		Start-Up Costs			Annual Ongoing Costs		
	Methods	Per Practice	Per Provider	Per Patient	Per Practice	Per Provider	Per Patient
Fleming, et al, 2016	Semi-structured interviews of related to staff time and financial resources to apply for and maintain NCQA level 3 recognition among a 57 practice health system in Texas	US$10 669^a^	US$2134				
Halladay, et al, 2016	Semi-structured interviews to collect data related to the staff time and financial resources expended when adopting new comprehensive primary care capabilities needed to apply for and maintain level 3 NCQA recognition among 4 practices in North Carolina		US$13 633	–		US$10 389	–
Magill, et al, 2015	Semi-structured interviews to estimate the costs that 16 practices incurred when delivering all capabilities related to comprehensive primary care among 20 practices in Utah and Colorado	–	–	–	–	US$104 799	US$52
Martsolf, et al, 2016	Semi-structured interviews to estimate the costs that 13 practices in Pennsylvania incurred when adopting new comprehensive primary care capabilities as part of a medical home demonstration program	US$30 991	US$9814	US$5	US$147 573	US$64 768	US$30
Nocon et al, 2016	Regression-based approach to estimate the marginal increase in operating costs as practices increased the number of comprehensive primary care capabilities among 669 safety net clinics	–	–	–	–	US$28,000	US$27
Patel et al, 2016	Compared staffing levels at 9 practices that the authors identified as experiencing medical home transformation to average staffing levels of primary care practices in the MGMA annual staffing survey	–	–	–	US$120,652	–	US$56
Shao et al, 2016	Regression-based approach to compare aggregated practice expenditures among 38 practices in New Orleans that received NCQA recognition, and 36 that did not, among safety net clinics participating in a federal primary care grant program	US$58 874			US$73 358	–	
Zuckerman et al, 2009	Regression-based approach to compare the operating costs of 11 practices that had high scores on the NCQA medical home recognition survey to 13 practices that had low scores	–	–	–	–	US$22 000	US$5

^a^Estimates represent initial accreditation application for a hypothetical 5-physician practice, renewal application was 4957.

### Overview of Findings

Fleming et al used semi-structured interviews to collect data related to staff time and other expenses incurred by a 57-practice medical group in Texas to apply for NCQA level 3 recognition, including developing the guidelines, protocols, and processes required by National Committee for Quality Assurance (NCQA) (ie, excluding any comprehensive primary care capabilities not required by NCQA).^15^ The authors estimated that the group spent US$1 508 503 overall in staff time for the initial application and US$346 617 for renewal. This is equivalent to US$10 669 (US$2134 per provider) for an initial application for a typical 5-provider practice and US$4957 (US$991 per provider) for renewal for a hypothetical 5-physician practice.

Halladay et al used semi-structured interviews to collect data related to the staff time and financial resources expended by 4 practices in North Carolina that were adopting new comprehensive primary care capabilities in order to apply for NCQA level 3 recognition.^13^ This study found that practices spent an average of US$13 633 per clinician in start-up costs and US$10 389 per clinician per year in ongoing costs to maintain new comprehensive primary care capabilities.

Magill et al used semi-structured interviews to estimate the costs that 16 practices in Utah and Colorado incurred delivering all capabilities related to comprehensive primary care.^14^ The average annual cost per clinician was approximately US$104 000 (or approximately US$52 per patient), and the average cost per clinical encounter was US$35 to adopt all capabilities consistent with the delivery of comprehensive primary care.

Martsolf et al also used semi-structured interviews to estimate the costs that 13 practices in Pennsylvania spent when adopting new comprehensive primary care capabilities as part of a medical home demonstration program. The median practice in this study spent US$30 991 per practice (US$9814 per clinician and US$8 per patient) on start-up costs and US$147 573 per year (US$64 768 per clinician and US$30 per patient) to maintain those capabilities.^12^

Nocon et al used a regression-based approach to estimate the marginal increase in operating costs as practices increased the number of comprehensive primary care capabilities among 669 safety net clinics. The authors measured comprehensive primary care using the Safety Net Medical Home (SNMH) scale, which uses a survey to count the number of comprehensive primary care capabilities adopted by a practice.^16^ Operating costs were collected via Uniform Data System reports that aggregate cost data reported by all health centers funded by the Health Resources and Services Administration’s Bureau of Primary Health Care. This study found that a 10-point increase in the SNMH score was associated with an additional US$27 950 in operating costs per provider (US$27 per patient) per year. In order to contextualize a 10-point change in SNMH, the authors reported that “The following 3 differences, in aggregate, would yield a 10-point higher total Patient-Centered Medical Home (PCMH) score for health center A: health center A is usually able to accommodate a same- or next-day appointment compared with never for health center B, health center A usually sends care reminders to patients compared with never for health center B, and health center A reports patient satisfaction surveys at the provider and group level, whereas health center B conducts no patient satisfaction reporting.”

Patel et al compared staffing levels at 9 practices that the authors identified as having undergone medical home transformation to average staffing at primary care practices included in the Medical Group Management Association (MGMA) annual staffing survey.^17^ The researchers estimated that a successful medical home should have 4.25 FTE staff per physician, a figure equivalent to 59% more staff per physician than the national average. The marginal costs of these greater staffing levels were estimated to be US$120 652 per physician (US$56 per patient per year, range US$45-US$77). This study focused only on staff time and did not include other cost categories, such as capital investments.

Analyzing data from New Orleans safety net clinics that participated in a federal primary care grant program, Shao et al used a regression-based approach to compare aggregated practice expenditures between 38 practices that received NCQA recognition and 36 that did not. Expenditures were estimated using program monitoring data for a large medical home demonstration program. Researchers found that practices that achieved NCQA recognition spent US$58 874 more than non-NCQA–accredited practices during the 1-year period of initial transformation and US$73 358 in the 3 years after transformation. This equates to US$38 and US$25 per patient visit, respectively.^11^

Finally, Zuckerman et al recruited 44 practices that had completed MGMA and American College of Physicians (ACP) Practice Management Checkup Tool in 2006. Zuckerman et al administered the 2008 version of the NCQA Physician Practice Connections-PCMH (PPC-PCMH) online self-assessment tool to these practices, obtaining usable responses from 35 of them. Of these, 11 practices had high scores on the NCQA survey (which the authors describe as being consistent with achieving NCQA’s Level 3 recognition levels) and 13 had low scores (consistent with level 1 recognition). The researchers then used a regression-based approach to compare the operating costs of high-scoring practices to those of low-scoring practices. Researchers estimated that high-scoring practices incurred on average US$22 000 more per FTE physician per year in operating expenses, or US$5 per patient per year.^18^

### Exploring Differences Across Estimates

Notable differences exist in the cost estimates across each of the studies that we have reviewed. First, studies measured different concepts. One study (Fleming et al) estimated the incremental costs of applying for NCQA medical home recognition without regard for baseline comprehensive capabilities within the practice, while others (Martsolf et al and Magill et al) estimated all comprehensive capabilities present in a practice regardless of NCQA application This may explain the lower costs observed in the former studies, compared to the latter.

Second, studies used different comparison groups to assess the marginal costs of comprehensive capabilities. Two of the studies (Patel et al and Shao et al) compared operating costs (either all operating costs or labor costs) at practices that have been designated as “medical homes” (either by the authors’ assessment or by NCQA recognition) to the average practice that had not been so designated. However, it is possible that an “average” practice not designated as a medical home could have adopted some comprehensive primary care capabilities. Other studies (eg, Magill et al) estimated the costs of all capabilities within a practice, implicitly making the comparison for these studies in practices that lacked any capabilities at all. This methodological difference may account for lower estimates in Shao et al compared to Magill et al.

In addition, 2 of the studies performed cross-sectional comparisons of relative costs between practices that had varying scores on composite scales of practice capabilities. Nocon et al compared practices that scored higher on the SNMH scale to those that scored lower, and Zuckerman et al compared practices that had high NCQA PPC-PCMH self-assessment scores to those with lower scores. By basing their comparison on a continuous score, Nocon et al used an approach that is not directly comparable to the other studies in this review (all of which base their comparisons on discrete categories of practices). The approach employed by Zuckerman et al, which calculated marginal costs using lack of NCQA recognition or nonparticipation in a medical home pilot as a reference point, is similarly noncomparable to the other studies in this review. Moreover, Zuckerman et al collected practice cost data that preceded assessment of medical home capabilities by approximately 2 years, rather than collecting contemporaneous cost and capability data. If “level-3” practices transformed to a greater extent between 2006 and 2008 than “level 1” practices (as measured in 2008), the costs of new capabilities detected on the PPC-PCMH would not have been captured, thereby underestimating the marginal costs of achieving level-3 scores (compared to level-1 scores).

## Methodological Limitations of Published Studies

The studies included in this literature review have limited generalizability. First, they focused on small numbers of practices in specific geographic areas (Pennsylvania, Utah, Colorado, North Carolina, and Texas),^12–15^ a convenience sample of practices chosen by the researchers,^19^ or on specific types of practices: safety net health centers^11^ or practices with NCQA recognition.^18^ As a result, these studies are unlikely to be representative of primary care practices nationwide. Second, although costs of comprehensive primary care capabilities are likely to vary based on patient and practice factors,^20^ none of the studies in our review attempted to estimate differences in costs across practices with patients at varying levels of complexity and illness burden. Only one study examined cost differences based on practice characteristics, finding higher costs per patient among small and independent practices^12^—though the sample sizes underlying this comparison were too small to allow meaningful statistical inference. Third, only 3 of the studies estimated startup costs associated with adopting comprehensive primary care capabilities. Accurate estimates of startup costs will be important to payers seeking to spur adoption of new capabilities among practices with limited access to capital.

Fourth, some studies measured comprehensive primary care capabilities in a steady state (ie, as a level of achievement),^14^ while others measured the costs of adopting new capabilities (ie, as practice transformation over time).^12^ As with startup costs, the difference between achievement and transformation is important to the purpose of the comprehensive primary care payments, that is, whether payers want to reimburse practices for all the capabilities that they have, as opposed to incentivizing practices to adopt new ones. Fifth, studies lacked a uniform method for measuring comprehensive primary care capabilities. Methods ranged from practice leader surveys and interviews to practice NCQA recognition levels.

## Primary Care Payment Considerations

Our findings raise important considerations for alternative payment models. The most common approach to paying practices for delivering comprehensive primary care—such as the approach used by CMS’ Comprehensive Primary Care+ program—is to pay practices a risk-adjusted per patient per month care management fee in addition to fee-for-service payments.^9^ The studies we reviewed provide some guidance on how to set these fees. Five of the studies estimated comprehensive primary care capability maintenance costs of US$2 to US$5 per patient per month,^12,16,19^ after excluding an outlier that only included NCQA-recognized practices (which estimated US$0.40 per patient per month).^18^ These estimates overlap the range of payments (US$3-US$8 per patient per month) offered by sponsors of recent medical home demonstrations.^9^ If care management fees are meant to reimburse practices for practices’ current levels of comprehensive primary care, these limited numbers of studies suggest that current per patient per month fees are likely set within a reasonable range. However, if payers want to encourage practices to shift even more of their resources away from face-to-face visits toward comprehensive primary care, they might need to consider a larger per patient per month fees. Studies using microsimulation models have attempted to estimate the size of comprehensive primary care payments that would incentivize practices to adopt comprehensive primary care capabilities.^21,22^ To estimate and account for effects on practice finances, these simulations require empirical estimates of the costs of comprehensive primary care capabilities. The studies we reviewed can provide such estimates.

In addition to per patient per month care management fees, some payers and policymakers have proposed a number of other approaches to constructing payments for comprehensive primary care, including: (1) enhanced fee for services payments, (2) additional codes for medical home activities, and (3) comprehensive capitated payments. The current literature has relatively little to say about payment amounts under these approaches. Two studies estimated the incremental per visit costs of comprehensive primary care, which could be used to estimate the payment levels for enhanced fee-for-service payments per visit or by payers to estimate capitated payments by including information about historic fee for service (FFS) payments. One study estimated that practices that have achieved medical home status have higher operating costs of US$25 per patient visit; another estimated that operating costs are US$1.40 more per visit when practices experience a 10% point increase on a 60-point medical home scale.^11,16^ Due to differences in how these studies measure comprehensive primary care, it is difficult to reconcile these estimates and translate them into enhanced fee-for-service payment rates. No studies included in our review directly addressed the issue of setting payment rates using additional codes for medical home activities and comprehensive capitated payments. Therefore, additional research is needed to better understand how such payments would be constructed and how associated rates would be set.

## Conclusion

In an effort to promote comprehensive primary care, CMS and other payers have adopted new payment models. However, payers have little empirical basis, should they wish to use data, for structuring payments and setting payment levels in these new models. Our review of the existing literature relevant to estimating these costs finds that practices incur substantial costs when adopting and maintaining comprehensive primary care capabilities. However, the heterogeneous designs and limited generalizability of published studies highlight the need for additional research, especially if payers wish to link their financial support for comprehensive primary care capabilities to the costs of these capabilities for primary care practices.
